# Genomic Prediction and Heritability Estimation for Daughter Pregnancy Rate in U.S. Holstein Cows Using SNP, Epistasis and Haplotype Effects

**DOI:** 10.3390/ijms26125687

**Published:** 2025-06-13

**Authors:** Ruifei Yang, Dzianis Prakapenka, Zuoxiang Liang, Yang Da

**Affiliations:** 1 Department of Animal Science, University of Minnesota, Saint Paul, MN 55108, USA; 2 College of Animal Science and Technology, Yunnan Agricultural University, Kunming 650201, China; 3 Department of Epidemiology and Health Statistics, School of Public Health, Fujian Medical University, University Town, No 1, Xue Yuan Road, Fuzhou 350108, China

**Keywords:** genomic prediction, epistasis, haplotype, prediction accuracy, SNP, daughter pregnancy rate, Holstein cows

## Abstract

The contributions of additive, dominance, haplotype, and epistasis effects up to the third order to the accuracy of predicting daughter pregnancy rate (DPR) phenotypic values and to the phenotypic variance in U.S. Holstein cows were investigated using five samples with 25,827–133,934 cows and 74,855–75,209 SNPs. Heritability estimates showed that only additive × additive (A × A) epistasis effects had nonzero heritability and all other second- and third-order epistasis effects had zero heritability, and hence A × A was the only epistasis effects included in the prediction models. Based on the results of the largest sample with 133,934 cows, genomic heritability estimate was 0.044–0.054 for additive heritability, 0.005 for dominance heritability, 0.011–0.022 for haplotype heritability, and 0.052–0.062 for A × A heritability. The combination of additive (A) and A × A effects was the best prediction model based on the prediction accuracy. This best model improved the prediction accuracy over the A-only model by 4.88%, and had total heritability of 0.099 as the summation of the additive and A × A heritability estimates. Dominance and haplotype effects had minor contributions (0.97–2.44%) to prediction accuracy in models without A × A effects but had no contribution to prediction accuracy when A × A was in the prediction model. The partition of A × A effects into inter- and intra-chromosome A × A effects showed that inter-chromosome A × A were mainly responsible for the A × A contributions to prediction accuracy and phenotypic variance. Sample size had a major impact on prediction accuracy and the sample of 90,000 cows or 81,000 cows per training population had peak prediction accuracies that were 32.70–35.85% higher than in the sample with 25,827 cows. The largest sample with 133,934 cows had the smallest variations in prediction accuracy and slightly lower average prediction accuracy than in the sample with 90,000 cows.

## 1. Introduction

Daughter pregnancy rate (DPR) is one of the most important fertility traits of U.S. Holstein cows that measures the rate a cow becomes pregnant during each 21-day period [1]. The DPR trait exhibits low additive heritability and large random variations, which complicates the identification and understanding of its genetic basis, as well as the application of this knowledge for improving trait performance [2]. Previous studies have identified many additive effects of DPR, but the number of significant effects was considerably fewer than those observed for production traits [3]. A recent large-scale study also identified some sharply negative recessive alleles for DPR when in homozygous status and it was recommended to eliminate heifers carrying any of those recessive genotypes [3]. For genetic improvement for all heifers and cows, genomic selection using genome-wide single nucleotide polymorphism (SNP) markers has been a routine method that provides genomic estimated breeding values based on the additive model for genetic selection [4]. Genomic prediction of DPR using complex genetic effects such as epistasis and haplotype effects should help understand the potential involvement of complex genetic mechanisms of DPR and may identify new prediction models with improved prediction accuracy. Global SNP epistasis effects are interaction effects between SNPs on the same and different chromosomes, whereas haplotypes effects of a chromosome segment may contain high-order interaction effects of the SNPs within the chromosome segment. Prediction models with epistasis effects [5,6,7,8,9,10] or haplotype effects [11,12,13,14,15,16] were reported to have increased the accuracy of genomic prediction. Mixed model methods and computing tools were developed to integrate additive, dominance, epistasis and haplotype effects for genomic prediction and heritability estimation. However, only limited studies using such integrated models for genomic prediction were reported [17,18], and research was unavailable on genomic prediction of DPR using epistasis and haplotype effects. In this study, we evaluate the contributions of epistasis and haplotype effects to the phenotypic variance and to the accuracy of predicting the DPR phenotypic values using epistasis and haplotype models separately and jointly. We used 50,606 U.S. Holstein cows (Sample 1) to identify the best epistasis model and then validated this best model using three larger samples: Sample 2 with 133,934 cows, Sample 4 with 70,000 cows and Sample 5 with 90,000 cows. We also used 25,827 cows (Sample 3) mainly for finding the best haplotype model among many candidate haplotype models. The best epistasis and haplotype models were then combined in the same integrated models that were evaluated by all samples.

## 2. Results and Discussion

### 2.1. Initial Prediction Model Based on Genomic Heritability Estimates

Genomic heritability estimates using Sample 1 with 50,606 cows under the full epistasis model with additive effects (A), dominance effects (D) and epistasis effects up to the third order were used for identifying the initial prediction model. The results showed that only additive × additive (A × A or AA) epistasis effects had nonzero heritability (0.066) among all types of epistasis effects (Table 1). Therefore, the only type of epistasis effects to be included in the initial prediction models were A × A epistasis effects. Under the A + AA model, the A × A heritability was 0.067, slightly higher than the 0.066 estimate from the full epistasis model. To gain a more detailed understanding of A × A effects, we partitioned A × A effects into intra-chromosome A × A effects (A × A^intra^) and inter-chromosome A × A effects (A × A^inter^). The heritability estimate from the A + AA^inter^ + AA^intra^ model was 0.048 for A × A^inter^ and 0.019 for A × A^intra^, showing that inter-chromosome A × A effects were the majority of the A × A effects, accounting for about 72% (=0.048/0.067) of the A × A variance.

The initial A + AA model identified in Sample 1 was compared to the haplotype models in separate models, and the epistasis and haplotype models were integrated into one model. The prediction accuracies of these models were compared with the two SNP models and the heritability estimates were obtained in most of the five samples. Results of these analyses will be summarized in the following sequence: prediction accuracy of the SNP models that served as the baseline comparison, the epistasis models, haplotype models, integrated models with both epistasis and haplotype effects, genomic heritability estimates, and impact of sample size on prediction accuracy.

### 2.2. Prediction Accuracies of Two SNP Models

The first step of evaluating prediction accuracy using validation studies under various models was to establish the prediction accuracies of the SNP models to serve as the baseline comparison with the accuracies of more complex prediction models. Two SNP models were evaluated for each sample, the additive model (A-model), and the additive and dominance model (A + D model). The average prediction accuracy of the A-model across the 10-fold validations was 0.162, 0.184, 0.201, 0.208 and 0.205 for Sample 3 with 25,827 cow, Sample 1 with 50,606 cows, Sample 4 with 70,000 cows, Sample 5 with 90,000 cows, and Sample 2 with 133,934 cows, respectively. For the A + D model, the accuracy over the A-model was −0.62% for Sample 3, 0.54% for Sample 1, 0% for Samples 4 and 5, and 0.97% for Sample 2, showing that dominance effects slightly increased the prediction accuracy over the A-model in Samples 1 and 2 (Table 2). As to be shown next, dominance effects had no contribution to prediction accuracy for DPR when A × A effects were in the prediction model. Therefore, most of the complex models were compared with the A-only model, which is also used in the current genomic evaluation of U.S. Holstein cows.

### 2.3. Prediction Accuracies of Epistasis Models

Validation studies using Sample 1 with 50,606 cows showed that the two epistasis models with and without dominance effects, A + D + AA and A + AA, had the same accuracy of predicting the DPR phenotypic values, indicating that dominance effects had no contribution to the prediction accuracy when A × A effects were in the prediction model. The average prediction accuracy of the A + AA and A + D + AA was 4.89% higher than the A-model (0.193 vs. 0.184) and was 4.32% higher than the A + D model (0.193 vs. 0.185). Validation studies using Sample 2 with 133,934 cows showed that the A + AA and A + D + AA models had the same average prediction accuracy of 0.215, confirming that dominance effects had no contribution to the prediction accuracy when A × A effects were in the prediction model. In Sample 2, the average prediction accuracy of 0.215 was 4.88% higher than the A-model (0.215 vs. 0.205) and was 3.86% higher than the A + D model (0.215 vs. 0.207) (Table 2 and Table S1). Given that dominance effects had no contribution to prediction accuracy when A × A effects were in the prediction model, all prediction models with A × A effects in other samples were not compared with the A + D model. For Sample 3 with 25,827 cows, Sample 4 with 70,000 cows and Sample 5 with 90,000 cows, the accuracy increase in the A + AA model over the A-model was 1.85%, 3.48% and 3.85%, respectively. Sample 5, with 90,000 cows, had the highest accuracy for the A-model and the A + AA model, 0.208 for the A-model, and 0.216 for the A + AA model. For Sample 2, with 133,934 cows, the largest sample in this study, the accuracy was 0.205 for the A-model and 0.215 for the A + AA model. The A-model of Sample 5 was 1.46% higher than in Sample 2, and the A + AA model of Sample 5 was 0.46% higher than in Sample 2.

**Figure 1:**
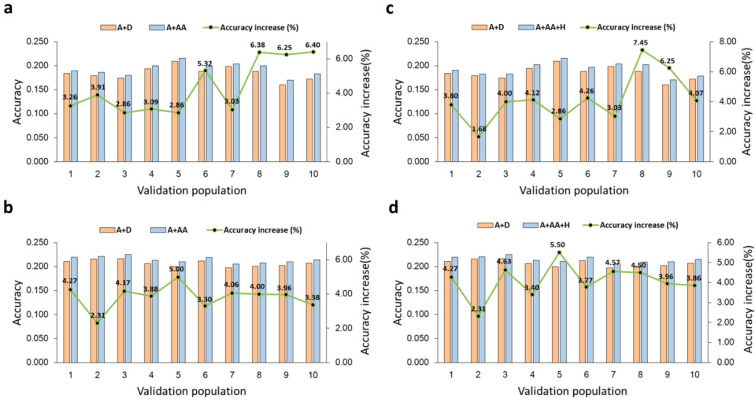
Prediction accuracy of DPR from 10-fold validations. (**a**) A + AA model in Sample 1 with 50,606 cows. (**b**) A + AA model in Sample 2 with 133,934 cows. (**c**) A + AA + H model in Sample 1 with 50,606 cows. (**d**) A + AA + H model in Sample 2 with 133,934 cows. The accuracy measure was the correlation between the GBLUP of total genetic values and the phenotypic values in each validation population. The percentage above each bar and the green trend line represent the accuracy increase due to A × A effects in each validation population.

Samples 1 and 2 had similar percentage accuracy increases (4.89% and 4.88%) due to A × A effects over the A-model, but Sample 2, with the largest sample size, had the smallest variations in prediction accuracy for all the prediction models in all samples (Table 2). Across the 10-validation populations, the accuracy increase was in the range of 2.31–5.00% in Sample 2 and 2.86–6.40% in Sample 1 (Figure 1a,b). The standard deviations of the prediction accuracies in Sample 2 were smaller than in Sample 1 for all prediction models, e.g., 0.004 in Sample 2 and 0.010 in Sample 1 for the A + AA model (Table 2).

### 2.4. Prediction Accuracy of Haplotype Models

We next evaluated the contribution of haplotype effects to prediction accuracy towards constructing integrated models with haplotype and epistasis effects. Two methods of haplotype blocking were used, a fixed number of SNPs (ranging from 2 to 20) and a fixed physical distance (from 50 Kb to 10 Mb). For each haplotype block size, four models (A + H, D + H, H, and A + D + H) were evaluated (Table S1). Each run used iterative solutions as well as matrix inversions and multiplications, generating a heavy computing load. To reduce computing difficulty, we used Sample 3 with 25,827 cows, a subset of Sample 1, for identifying the best haplotype model using 5-fold validations. Once the best haplotype model was identified, this model was re-evaluated using 10-fold validations to be consistent with the 10-fold validations for all other models and sample sizes. The results showed the best haplotype model was the A + H or A + D + H model for 7.5 Mb block size with 1.23% accuracy increase over the A + D model (0.164 vs. 0.162), where each 7.5 Mb block was treated as a ‘locus’ and each haplotype within the haplotype block as an ‘allele’, as defined by the multi-allelic haplotype model. However, dominance effects had no contribution to prediction accuracy when haplotype effects were in the prediction model. For this reason, the A + H model with 7.5 Mb haplotype blocks was considered the best haplotype model to be integrated with the epistasis model. For this A + H model with 7.5 Mb haplotype blocks, Sample 1 with 50,606 cows improved the prediction accuracy over the A-model by 2.17% (0.188 vs. 0.184) and Sample 2 with 133,934 cows improved the prediction accuracy over the A-model by 2.44% (0.210 vs. 0.205). Compared to the A + AA model, the A + H model had similar variations in prediction accuracy in terms of standard deviation of the accuracies across the 10 validation populations in each sample (Table 2). However, a notable difference between the A + AA and A + H models was that the A + H model had the highest prediction accuracy for one validation population in each sample, 7.45% in the eighth validation population of Sample 1 (Figure 1c) and 5.50% in the fifth validation population of Sample 2 (Figure 1d).

### 2.5. Accuracy of Integrated Models with Epistasis and Haplotype Effects

Each integrated model combines epistasis and haplotype effects, resulting in the A + AA + H model or A + D + AA + H model, where H is the haplotype effects of 7.5 Mb haplotype blocks. Validation results of Sample 1 with 50,606 cows showed that the A + AA + H model had slightly lower accuracy (0.192) than that of the A + AA model (0.193). The A + D + AA + H model had the same accuracy (0.193) as that of the A + D + AA model. Validation results of Sample 2 with 133,934 cows showed that the A + AA + H model and the A + AA model had the same prediction accuracy (0.215) (Table 2). Since the sample size of Sample 2 was 2.65 times as large as that of Sample 1, we used the results of Sample 2 to conclude that haplotypes had no contribution to prediction accuracy for DPR. The slight difference in prediction accuracy between A + AA + H and A + D + AA + H models was likely due to random variations. Combining all validation results, dominance and haplotype effects had no contribution to prediction accuracy, the A + AA model was the best prediction model, and A × A effects were more important than haplotype effects for predicting the phenotypic values of DPR, noting that a human observed the opposite: haplotype effects tended to be more important than epistasis effects [18].

### 2.6. Contributions of Intra- and Inter-Chromosome A × A Effects to Prediction Accuracy

To evaluate the contributions of AA^intra^ and AA^inter^ to prediction accuracy, six models involving AA^intra^ and AA^inter^ were evaluated for prediction accuracy using 10-fold validations using Sample 1 with 50,606 cows (Table 3). Under the A + AA^intra^ + AA^inter^ model, the prediction accuracy was the same as the accuracy of the A + AA model (0.193), with 4.89% accuracy increase over the A-model. Removing AA^intra^, the A + AA^inter^ model had an average prediction accuracy of 0.192, only slightly (0.54%) lower than the accuracy of the A + AA model (0.193). Removing AA^inter^, the A + AA^intra^ model had an average prediction accuracy of 0.189, substantially (2.18%) lower than the accuracy of the A + AA model. These results of the two separate A + AA^inter^ and A + AA^intra^ models indicated that both AA^intra^ and AA^inter^ affected the prediction accuracy but AA^inter^ nearly had the same accuracy as that of all A × A effects, and AA^intra^ only had a minor contribution to the prediction accuracy.

The addition of haplotypes to the models with AA^intra^ and AA^inter^ provided an understanding about the lack of haplotype contributions to prediction accuracy when A × A effects were in the prediction model. The A + AA^inter^ + AA^inta^ + H had slightly lower prediction accuracy than the A + AA^inter^ + AA^inta^ model without haplotypes, 0.192 vs. 0.193. Removing AA^intra^, the A + AA^inter^ + H model had the same accuracy of 0.192 as that of the A + AA^inter^ + AA^inta^ + H or that of the A + AA^inter^ model, indicating that haplotypes had no contribution to the prediction accuracy when AA^inter^ was in the prediction model, indicating confounding between AA^inter^ and H. This confounding result was nonintuitive, given that AA^inter^ involves interactions between two loci on different chromosomes, whereas H may involve interactions among some or all loci in the same chromosome region. Removing AA^inter^, the A + AA^intra^ + H model had a prediction accuracy of 0.190, slightly higher than the 0.189 accuracy of the A + AA^intra^ model with haplotypes, indicating that haplotypes slightly increased the prediction accuracy when AA^intra^ was in the prediction model. However, AA^intra^ only had a minor contribution, and haplotypes had no contribution to prediction accuracy when AA^inter^ effects were in the prediction model. These accuracy results (Table 3) along with the heritability estimates of AA^inter^ and AA^inta^ (Table 1) showed that inter-chromosome A × A effects were the primary contributor to the prediction accuracy among AA^inter^, AA^inta^ and H for DPR in Holstein cows. This result should be interpreted as new evidence of the diverse responses to complex epistasis and haplotype effects from different traits in different species.

### 2.7. Genomic Heritability Estimates from 10-Fold Validations

Genomic heritability estimates under different prediction models provided an understanding about the contributions of different effect types to the phenotypic variance, and such estimates from the 10-fold validations proved assessments about the variations in the heritability estimates from different validations. Genomic heritability estimates were obtained for eleven models in Sample 1 and five models in Sample 2 (Table 4). For the same models, Sample 2 with 133,934 cows and lower heritability estimates than those from Sample 1 with 50,606 cows. The additive heritability estimate was the same for both SNP models (A-model and A + D), 0.044 in Sample 2 and 0.054 in Sample 2. The dominance heritability under the A + D model was 0.005 from Sample 2 and 0.006 from Sample 1, yielding total SNP heritability of 0.050 in Sample 2 and 0.060 in Sample 1. The A × A heritability under the A + AA model was 0.062 from Sample 2 and 0.067 from Sample 1, and the haplotype heritability estimate under the A + H model was 0.022 from Sample 2 and 0.030 from Sample 1. Heritability estimates of additive and dominance effects had the same standard deviation of 0.001 from the 10-fold validations in both samples, but Sample 2 had smaller standard deviations than in Sample 1 for heritability estimates of A × A and haplotype effects (Table 4). 

Genomic heritability estimates from prediction models involving AA^intra,^, AA^inter^ and haplotype effects (H) using Sample 1 with 50,606 cows provided an understanding about the confounding between A × A and haplotypes effects of DPR (Table 4). Without haplotypes (H) in the model, the heritability estimate was 0.047 for AA^inter^ and 0.020 for AA^intra,^ under the A + AA^inter^ + AA^intra^ model. Adding haplotypes to the model, the heritability estimates reduced to 0.035 and 0.014 for AA^intra^ and AA^inter^, respectively, the additive heritability estimate reduced from 0.046 to 0.039, and the haplotype heritability estimate was 0.019. These results showed that the reduction in heritability estimates due to haplotypes was 0.007 for additive heritability, 0.012 for AA^inter^ heritability, and 0.006 for AA^intra,^ heritability, indicating that AA^inter^ had the largest share of the heritability estimates redistributed to haplotype heritability estimates while some additive and AA^intra^ heritability estimates were also redistributed to the haplotype heritability estimate, consistent with the results of prediction accuracies indicating that haplotype effects were mostly confounded with AA^inter^. The comparison between the four models with AA^inter^ or AA^intra^ effects provided further confirmation that haplotype effects were mostly confounded with AA^inter^ effects.

**Figure 2:**
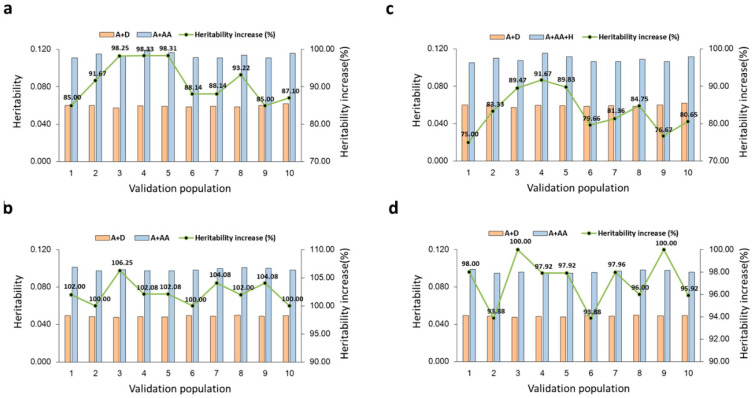
Estimates of total heritability of DPR from 10-fold validations. (**a**) A + AA model in Sample 1 with 50,606 cows. (**b**) A + AA model in Sample 2 with 133,934 cows. (**c**) A + AA + H model in Sample 1 with 50,606 cows. (**d**) A + AA + H model in Sample 2 with 133,934 cows. The accuracy measure was the correlation between the GBLUP of total genetic values and the phenotypic values in each validation population. The percentage above each bar and the green trend line represent the heritability increase due to A × A effects in each validation population.

Across the 10 validations, estimates of the total SNP heritability hardly had visible variations in both samples (Figure 2). In contrast, the estimates of the A × A heritability (Table 4) and the total heritability of the A + AA model (Figure 2a) had larger variations in Sample 1 than in Sample 2 (Figure 2b) with standard deviation in Sample 1 that was three times as large as in Sample 2 (Table 4). The estimates of haplotype heritability under the A + AA + H model had similar patterns as the A × A heritability estimates (Figure 2c,d). Since both samples had similar variations in SNP heritability estimates but only Sample 2 had similar variations for SNP, A × A and haplotype heritability estimates, the sample size of Sample 1 was adequate for SNP heritability estimation, but only Sample 2 had a desirable sample size for heritability estimation of A × A and haplotype effects for DPR.

### 2.8. Sample Size and Prediction Accuracy

**Figure 3:**
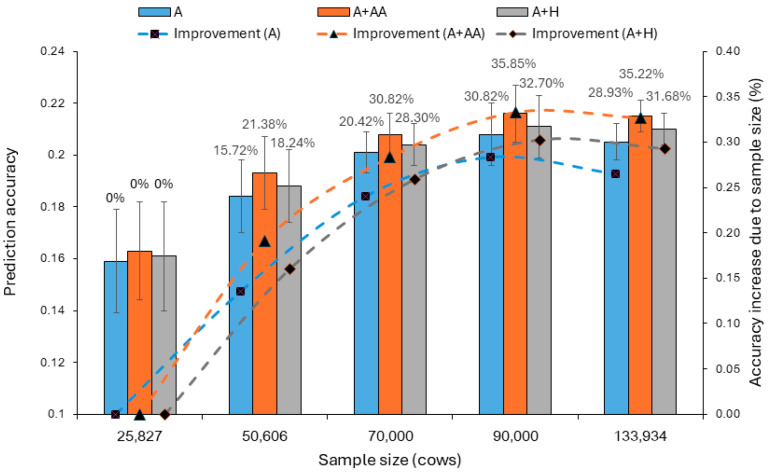
Relationship between prediction accuracy and sample size from 10-fold validations. Prediction accuracy increased as sample size increased until the sample size reached 90,000 or more, but the largest sample had the smallest variations in prediction accuracy. ‘Improvement’ and ‘accuracy increase’ indicate the accuracy increase relative to the sample with 25,827 cows.

In this study, sample size had the largest impact on the prediction accuracy in the validation populations (Figure 3). Prediction accuracy increased as the sample size increased from 25,827 cows (Sample 3) to 50,606 cows, (Sample 1), to 70,000 cows (Sample 4), and to 90,000 cows (Sample 5) where prediction accuracy appeared to have peaked. From 90,000 cows to 133,934 cows (Sample 2), prediction accuracy slightly decreased for three models: 0.208 in Sample 5 vs. 0.205 in Sample 2 for the A-model, 0.216 vs. 0.215 for the A + AA model and 0.211 vs. 0.210 for the A + H model, but Sample 2 had smaller variations in prediction accuracy than those in Sample 5, with standard deviations that were 41–45% smaller than in Sample 5 (Table 2). The accuracy increase in the largest sample (Sample 2) over the accuracy of the smallest sample (Sample 3) was 28.93% for the A-model, 35.22% for the A + AA model, and 31.68% for the A + H model. These differences were much larger than the differences between different models within the same sample, e.g., the 4.88% accuracy increase of the A + AA model over the accuracy of the A-model in Sample 2.

The large impact of sample size on the prediction accuracy and variations in prediction accuracy for all prediction models in this study suggested that the sample size should be sufficiently large to achieve maximum accuracy and minimal variations in prediction accuracy. For prediction accuracy only, without considering variations in prediction accuracy, the optimal sample size is the smallest sample size achieving the maximum prediction accuracy and involves a known factor of statistical limit and an unknown factor of genetic limit.

The statistical limit is what could be characterized as ‘n–m’ confounding. For the additive model, the genomic relationship matrix (GRM) is singular when the number of individuals (n) is greater than the number of SNPs (m) [19]. Assuming n independent individuals with respect to SNP genotypes and m independent SNPs where no SNP could be expressed in terms of any other SNPs, the rank of the GRM is the smaller of the n and m. Because of the singularity of the n×n GRM when n > m, the extra individuals beyond m have no contribution to prediction accuracy [20,21]. Therefore, m sets the statistical limit to the sample size of the training population where all individuals have independent SNP information for genomic prediction.

The ranks of additive and dominance GRMs in Sample 3 with 25,827 cows, Sample 1 with 50,606 cows and Sample 2 with 133,934 cows (Table 5) confirmed this statistical limit for the two SNP models. For these three samples, the number of SNPs (m) was 75,209 for Samples 1 and 3, and was 74,855 for Sample 2. For Samples 1 and 3, the n–m structure was n < m so that each SNP GRM was expected to be singular with the rank of n. The observed rank in Samples 1 and 3 was less than, but close to, n, the number of cows. In Sample 3, with 25,827 cows, the rank was 25,817 for the additive GRM and 25,818 for the dominance GRM; and in Sample 1 with 50,606 cows, the rank was 50,594 for the additive GRM and 50,595 for the dominance GRM. The reason for the ranks to be close to but less than the number of cows should be the presence of cows with identical SNP genotypes such as identical twins or clones. Sample 2, with 133,934 cows (120,540 cows per training population in the 10-fold validations), had the n–m structure of n > m, opposite to the n < m structure of Samples 1 and 3, and the rank of each SNP GRM of Sample 2 was expected to be m = 74,855. The observed rank in Sample 2 was 74,853 for both additive and dominance GRMs, two SNPs fewer than 74,855, indicating that two SNPs likely were in complete linkage disequilibrium with two other SNPs. Note that singular GRMs do not cause any problem to the methods of genomic prediction and heritability estimation in this study because the multifactorial mixed model methods used in this study do not require inverting GRMs [17]. 

The observed ranks of SNP GRMs from all three samples confirmed that the rank of each SNP GRM was the smaller of n and m. Given the statistical limit of n–m confounding due to the singular GRM, Sample 2, with 133,934 cows (120,540 cows per training population in the 10-fold validations), and Sample 5, with 90,000 cows (81,000 cows per training population in the 10-fold validations), were expected to have the same prediction accuracy for the SNP models. However, the statistical limit due to n–m confounding was expected to be nonexistent for epistasis and haplotype models, because epistasis and haplotype effects typically have huge numbers of levels [14,17] so that each n × n epistasis or haplotype GRM is expected to be of full rank (=n) virtually in all practical situations without individuals with identical SNP genotypes. The observed rank of Sample 2 with 133,934 cows was 133,896 for any of the seven epistasis GRMs and 133,897 for the haplotype additive GRM (Table 5). The small differences between n = 133,934 and the ranks were again likely due to the presence of cows with identical SNP genotypes such as identical twins and clones. These results were consistent with the expectation that each epistasis or haplotype GRM should be of full rank without individuals with identical SNP genotypes. Because of the full-rank GRMs, the statistical limit arising from singular GRMs did not exist for the epistasis and haplotype models. However, the increase in sample size from 90,000 to 133,934 cows did not increase the accuracy for the epistasis and haplotype models. We hypothesize that genetic limit was the likely reason for the lack of accuracy increase beyond a certain sample size. For DPR in this study, the sample size of 81,000 cows per training population appeared to have achieved the full genetic potential for prediction accuracy of the epistasis and haplotype models investigated for DPR in this study. Although the average prediction accuracy did not increase, variations in prediction accuracy continued to shrink as the sample size increased for all prediction models (Figure 3). Therefore, large samples still should have an advantage for stable prediction accuracy, even if the average prediction accuracy peaked with a smaller sample size.

### 2.9. Prediction Accuracy in Training Populations

The relationship between sample size and the prediction accuracy in the training population was the opposite to that of validation populations: prediction accuracy in the training population decreased as the sample size increased. The prediction accuracy of the A-model in the training population was 0.383, 0.327, 0.305, 0.304, and 0.289 for sample sizes of 25,827, 50,606, 70,000, 90,000, and 133,934 cows, respectively (Table 2). The difference between the training and validation populations is in the use of the phenotypic values for calculating GBLUP: a training population calculates GBLUP using phenotypic observations of all individuals in the population whereas the phenotypic observations of each validation population are omitted when calculating GBLUP to predict the validation individuals. This difference should explain the opposite relationship between the sample size and the prediction accuracy in training and validation populations: more related individuals increased the prediction accuracy when the phenotypic observations of the individuals being predicted were unavailable for calculating GBLUP but decreased the prediction accuracy when the phenotypic observations of the individuals being predicted were used for calculating GBLUP.

Within each sample or for a given sample size, the accuracy of a prediction model is a measure of how well the model fits the data and is a measure of the relevance of the genetic effects in the prediction model to the phenotypic values. Across the five samples, validation results in the training populations showed that dominance, epistasis and haplotype effects were all relevant to the DPR phenotypic values. The accuracy increase over the A-model was 3.93–8.86% by the A + D model, 17.43–20.63% by the A + H model, and 65.90–81.65% by the A + AA model (Table 2). These results in the training populations were consistent with those in the validation populations, showing that A × A effects had greater influence than haplotype and dominance effects on the DPR phenotypic values.

## 3. Materials and Methods

### 3.1. Holstein Populations and Genotyping Data

Five samples were used for genomic prediction: Sample 1 with 50,606 cows, Sample 2 with 133,934 cows, Sample 3 with 25,827 cows as a subset of Sample 1 for identifying the best haplotype model, and Samples 4 and 5 with 70,000 and 90,000 cows, respectively, for additional evidence about the impact of sample size on prediction accuracy (Table 6). The DPR phenotypic values were the phenotypic residuals after removing fixed non-genetic effects available from the December 2022 U.S. Holstein genomic evaluation data produced by Council on Dairy Cattle Breeding (CDCB). Sample 1 was used for identifying the best prediction model, a process involving the analysis of many candidate models. Sample 2 was used as a large sample validation of the best prediction model identified by Sample 1, including genomic heritability estimation and prediction accuracy. The SNP genotypes were from 32 SNP chips with various densities and were imputed to 78,964 SNPs (80K) via FindHap algorithm [22] as a routine procedure for genomic evaluation by Council on Dairy Cattle Breeding CDCB. The SNP genotyping quality control [4] included the call rate, parent–progeny conflicts, sex verification using X-specific SNPs, and Hardy–Weinberg equilibrium. Furthermore, we selected cows with a minimum SNP density of 40K original SNPs for Sample 1 and 30K for Sample 2 to minimize potential imputing errors from lower densities to the 80K SNPs. A minor allele frequency (MAF) threshold of 5% was applied for SNP filtering, resulting in final SNP sets of 74,855–75,209 SNPs for the five samples (Table 6).

To reduce costly computational time for haplotype model evaluation, the genomic predictions of the haplotype model were based on a subset of 50,606 cows from Sample 1, from which 25,827 individuals were randomly extracted. The haplotype GRMs were constructed using various blocking methods. These methods included distance-based blocks ranging from 100 Kb, 500 Kb, 1000 Kb, 1.5 Mb, 2 Mb, up to 10 Mb, and SNP number blocks varying from 2 SNPs to 20 SNPs. For the SNP number-based blocks, we employed a sliding window approach for comparison, with a step size of one SNP. Regarding the distance-based blocks, to mitigate the computational burden associated with constructing GRMs for numerous haplotype datasets across various window sizes using the sliding window method, we opted to build only one GRM corresponding to the block size that demonstrated the highest prediction accuracy among the haplotype data using a distance-based haplotype without a sliding window. The sliding window for this method was also set to one SNP.

### 3.2. Mixed Model for GBLUP and GREML

Genomic best linear unbiased prediction (GBLUP) was used to assess the prediction accuracy of genomic estimated breeding values, whereas genomic restricted maximum likelihood estimation (GREML) was applied to determine the heritability levels of different genetic effects through a multifactorial mixed model [17]. The effects analyzed by GBLUP and GREML included SNP additive and dominance effects, haplotype effects, as well as global epistasis effects up to the third order. The mixed model is described as follows:

(1) $$y=Xµ+Zg+e=Xµ+Z\sum_{i=1}^{f}u_{i}+e$$

(2) $$g=\sum_{i=1}^{f}u_{i}$$

(3) $$V=ZGZ^{\prime }+\sigma_{e}^{2}I_{N}=Z\left(\sum_{i=1}^{f}G_{i}\right)Z^{\prime }+\sigma_{e}^{2}I_{N}=Z\left(\sum_{i=1}^{f}\sigma_{i}^{2}S_{i}\right)Z^{\prime }+\sigma_{e}^{2}I_{N}$$

(4) $$G=\mathrm{Var}\left(g\right)=\mathrm{Var}\left(\sum_{i=1}^{f}u_{i}\right)=\sum_{i=1}^{f}\mathrm{Var}(u_{i})=\sum_{i=1}^{f}\sigma_{i}^{2}S_{i}$$

where y= N × 1 column vector of phenotypic observations, X= N × 1 column vector of **1**′s as the model matrix for µ, µ = mean of the phenotypic values, Z= N × n incidence matrix allocating phenotypic observations to each individual, N = number of observations, n = number of individuals and N = n when every individual has one observation, e= N × 1 column vector of random residuals, $\sigma_{e}^{2}$ = the residual variance, $u_{i}=$ n × 1 column vector of the genetic effects of the $i^{\mathrm{th}}$ effect type,$\;\sigma_{i}^{2}$ = variance of the genetic effects of the $i^{\mathrm{th}}$ effect type, $S_{i}=$ n × n genomic relationship matrix of the $i^{\mathrm{th}}$ effect type, $g=\sum_{i=1}^{f}u_{i}\;$= n × 1 column vector of the total genetic values, and f = number of effect types. In this study, subscript i = 1 to 9 in Equations (1)–(4) represent SNP and epistasis effects (Table 1), and subscript i = 10 represents haplotype additive effect. Additionally, we partitioned global A × A epistasis effects into intra-chromosome and inter-chromosome A × A effects, as outlined in Table 1. The epistasis GRMs were calculated using the genomic version of Henderson’s Hadamard products [23,24,25,26], which were approximate epistasis GRMs but practically had the same results as the exact epistasis GRMs [27] according to our previous evaluations [6,17,18]. The EPIHAP program (https://github.com/AnimalGene/EPIHAP) (accessed on 12 June 2025) was used for the data analysis of Samples 1 and 3, whereas the data analysis of Samples 2, 4 and 5 used the parallel computing version of EPIHAP named EPIHAPMPI (https://github.com/dprakapenka/EPIHAPMPI) (accessed on 12 June 2025) was used for. The figures in this article were made using Excel.

### 3.3. Evaluation of Prediction Accuracy Using Cross-Validation

A 10-fold cross-validation approach was implemented to assess the prediction accuracies of various models. Individuals with phenotypic data were randomly separated into 10 different validation groups. For each validation group, predictions were made using a training set and the other nine validation groups. The first nine groups maintained an equal size of samples, while the tenth group contained the remaining individuals. The phenotypic values of validation cows were excluded from each validation population when calculating GBLUP for the training and validation cows in the validation population. To evaluate the accuracy of the observed predictions, data from 50,606 or 25,827 (for haplotype model evaluation) individuals were examined utilizing the corresponding GBLUP model for Sample 1, and we further evaluated the best prediction model from 133,934 individuals for Sample 2. The accuracy of predicting the phenotypic values in the validation populations was calculated as the Pearson correlation between the GBLUP of the total genetic values ($\hat{g}$) and the phenotype values (y) of the validation cows, $\;\hat{R}=corr(\hat{g},\;y$), averaged across all validation datasets [28].

### 3.4. Initial Selection of Epistasis Effects for Prediction Models

The initial selection of epistasis models was designed to exclude effect types that contribute minimally, or not at all, to phenotypic variance from further evaluation concerning prediction accuracy. Each effect type was required to have a heritability estimate exceeding 0.5% to be included in the prediction model for subsequent analysis.

## 4. Conclusions

Heritability estimates and the accuracy of genomic prediction showed that A × A epistasis effects were the only type of epistasis effects contributing to the prediction accuracy and phenotypic variance of DPR among all epistasis effects up to the third order. Haplotype effects contributed to the prediction accuracy and phenotypic variance in the absence of A × A effects but had no contribution when A × A effects were in the prediction model. These findings demonstrate that both epistasis and haplotype effects affected DPR but A × A effects were predominant over haplotype effects for prediction accuracy.

## Figures and Tables

**Table 1:** Heritability estimates of the full model with SNP and epistasis effects up to the third order.

Sample 1 (n = 50,606)	Subscript in Equations (1) and (2)	A	A + D	Full Model	A + AA	A + AA^inter^ + AA^intra^
A	1	0.053	0.053	0.047	0.047	0.045
D	2	-	0.006	0.004	-	-
A × A	3	-	-	0.066	0.067	-
A × A^inter^	3	-	-	-	-	0.048
A × A^intra^	4	-	-	-	-	0.019
A × D	4	-	-	0.000	-	-
D × D	5	-	-	0.000	-	-
A × A × A	6	-	-	0.000	-	-
A× D × D	7	-	-	0.000	-	-
A × D × D	8	-	-	0.000	-	-
D × D× D	9	-	-	0.000	-	-
Total Heritability		0.053	0.059	0.116	0.114	0.112
Initial Model		-	-	-	A + AA	A + AA^inter^ + AA^intra^
**Sample 2** **(n = 133,934)**	**Subscript in Equations (1) and (2)**	**A**	**A + D**	**Full Model**	**A + AA**	
A	1	0.044	0.044	0.037	0.036	
D	2	-	0.005	0.003	-	
A × A	3	-	-	0.060	0.061	
A × D	4	-	-	0.000	-	
D × D	5	-	-	0.000	-	
A × A × A	6	-	-	0.000	-	
A× D × D	7	-	-	0.000	-	
A × D × D	8	-	-	0.000	-	
D × D× D	9	-	-	0.000	-	
Total heritability		0.044 ± 0.000	0.049 ± 0.002	0.099	0.097	
Initial model		-	-	-	A + AA	

A is additive effects. D is dominance effects. A × A, A × D and D × D effects are second-order (pairwise) epistasis effects. A × A × A, A× D × D, A × D × D and D × D× D are third-order epistasis effects. AA with superscripts inter and intra are the inter- and intra-chromosome A × A epistasis effects. Hyphen ‘-’ indicates effect type not included in the prediction model.

**Table 2:** Accuracy of different models for predicting DPR phenotypic values from 10-fold validations.

	Prediction Accuracy		Accuracy Increase over A-Model (%)	
Model	T	V	T	V
	SNP model			
A (n = 25,827)	0.383 ± 0.004	0.162 ± 0.013	0.00	0.00
A + D (n = 25,827)	0.415 ± 0.028	0.161 ± 0.012	8.36	−0.62
A (n = 50,606)	0.327 ± 0.002	0.184 ± 0.014	0.00	0.00
A + D (n = 50,606)	0.356 ± 0.004	0.185 ± 0.014	8.86	0.54
A (n = 70,000)	0.305 ± 0.001	0.201 ± 0.008	0.00	0.00
A + D (n = 70,000)	0.317 ± 0.002	0.201 ± 0.008	3.93	0.00
A (n = 90,000)	0.304 ± 0.002	0.208 ± 0.012	0.00	0.00
A + D (n = 90,000)	0.322 ± 0.003	0.208 ± 0.012	5.92	0.00
A (n = 133,934)	0.289 ± 0.001	0.205 ± 0.007	0.00	0.00
A + D (n = 133,934)	0.310 ± 0.002	0.207 ± 0.007	7.27	0.97
	Global epistasis model			
A + AA (n = 25,827)	0.649 ± 0.075	0.165 ± 0.014	69.45	1.85
A + AA (n = 50,606)	0.594 ± 0.010	0.193 ± 0.014	81.65	4.89
A + D + AA (n = 50,606)	0.601 ± 0.011	0.193 ± 0.014	83.79	4.89
A + AA (n = 70,000)	0.506 ± 0.007	0.208 ± 0.008	65.90	3.48
A + AA (n = 90,000)	0.520 ± 0.001	0.216 ± 0.011	71.05	3.85
A + AA (n = 133,934)	0.520 ± 0.004	0.215 ± 0.006	79.93	4.88
A + D + AA (n = 133,934)	0.524 ± 0.004	0.215 ± 0.006	81.31	4.88
	Haplotype model			
A + H (n = 25,827)	0.462 ± 0.016	0.164 ± 0.014	20.63	1.23
A + H (n = 50,606)	0.406 ± 0.004	0.188 ± 0.014	14.04	2.17
A + H (n = 70,000)	0.362 ± 0.003	0.204 ± 0.008	18.69	1.49
A + H (n = 90,000)	0.357 ± 0.002	0.211 ± 0.012	17.43	1.44
A + H (n = 133,934)	0.341 ± 0.002	0.210 ± 0.006	17.99	2.44
	Integrated model			
A + AA + H (n = 25,827)	0.633 ± 0.076	0.166 ± 0.014	65.27	2.47
A + AA + H (n = 50,606)	0.572 ± 0.013	0.192 ± 0.014	74.92	4.35
A + AA + H (n = 70,000)	0.487 ± 0.007	0.208 ± 0.008	59.67	3.48
A + AA + H (n = 90,000)	0.505 ± 0.011	0.216 ± 0.011	66.12	3.85
A + AA + H (n = 133,934)	0.509 ± 0.004	0.215 ± 0.006	76.12	4.88

A is SNP additive values. D is SNP dominance values. H is haplotype additive values. AA is A × A values. T is training populations and V is validation populations. The number after “±” is the standard deviation across the 10-fold validations.

**Table 3:** Accuracy of intra- and inter-chromosome A × A epistasis effects for predicting DPR phenotypic values from 10-fold validations in Sample 1 with 50,606 cows.

	Prediction Accuracy		Accuracy Increase over A-Model (%)	
Model	T	V	T	V
A + AA^inter^ + AA^intra^	0.586 ± 0.010	0.193 ± 0.013	79.20	4.89
A + AA^inter^	0.589 ± 0.010	0.192 ± 0.014	80.12	4.35
A + AA^intra^	0.464 ± 0.009	0.189 ± 0.013	41.89	2.72
A + AA^inter^ +AA^inta^ + H	0.568 ± 0.012	0.192 ± 0.013	59.55	4.35
A + AA^inter^ + H	0.569 ± 0.012	0.192 ± 0.014	74.01	4.35
A + AA^intra^ + H	0.477 ± 0.009	0.190 ± 0.013	45.87	3.26

A is SNP additive values. D is SNP dominance values. H is haplotype additive values. AA is A × A values. T is training populations and V is validation populations. The number after “±” is the standard deviation across the 10-fold validations.

**Table 4:** Genomic heritability estimates as averages from 10-fold validations in two samples.

Model	Sample 1 with 50,606 Cows						
	A	D	HH	AA	AA^inter^	AA^intra^	Total
A	0.054 ± 0.001	-	-	-	-	-	0.054 ± 0.001
A + D	0.054 ± 0.001	0.006 ± 0.001	-	-	-	-	0.060 ± 0.001
A + AA	0.047 ± 0.001	-	-	0.067 ± 0.003	-	-	0.114 ± 0.003
A + H	0.040 ± 0.002	-	0.030 ± 0.002	-	-	-	0.070 ± 0.001
A + AA^inter^	0.047 ± 0.001	-	-	-	0.065 ± 0.003	-	0.112 ± 0.003
A + AA^intra^	0.047 ± 0.002	-	-	-	-	0.036 ± 0.003	0.083 ± 0.002
A + AA^inter^ + H	0.040 ± 0.002	-	0.021 ± 0.003	-	0.047 ± 0.005	-	0.108 ± 0.003
A + AA^intra^ + H	0.039 ± 0.002	-	0.023 ± 0.002	-	-	0.024 ± 0.002	0.086 ± 0.002
A + AA^inter^ + AA^intra^	0.046 ± 0.001	-	-	-	0.047 ± 0.004	0.020 ± 0.003	0.112 ± 0.003
A + AA^inter^ +AA^inta^ + H	0.039 ± 0.002	-	0.019 ± 0.003	-	0.035 ± 0.004	0.014 ± 0.003	0.108 ± 0.003
A + H + AA	0.040 ± 0.002	-	0.021 ± 0.003	0.049 ± 0.005	-	-	0.109 ± 0.003
	**Sample 2 with 133,934 cows**						
A	0.044 ± 0.001	-	-	0.044 ± 0.001			0.044 ± 0.001
A + D	0.044 ± 0.001	0.005 ± 0.001	-	-			0.049 ± 0.001
A + AA	0.037 ± 0.001	-	-	0.062 ± 0.001			0.099 ± 0.001
A + H	0.034 ± 0.001	-	0.022 ± 0.000	-	-	-	0.056 ± 0.001
A + H + AA	0.033 ± 0.001	-	0.011 ± 0.000	0.052 ± 0.001			0.096 ± 0.001

A is SNP additive values. D is SNP dominance values. H is haplotype additive values. AA is A × A values. AA with subscripts inter and intra are the inter-chromosome and intra-chromosome A × A epistasis values. The number after ‘±’ is the standard deviation of the training populations across the 10-fold validations.

**Table 5:** Rank of genomic relationship matrix (GRM) for each effect type from three samples.

	Sample 3 (m = 75,209)		Sample 1 (m = 75,209)		Sample 2 (m = 74,855)	
GRM	No. Cows (n)	Rank	No. Cows (n)	Rank	No. Cows (n)	Rank
A ($S_{1}$)	25,827	25,817	50,606	50,594	133,934	74,853
D ($S_{2}$)	25,827	25,818	50,606	50,595	133,934	74,853
AA ($S_{3}$)	25,827	25,818	50,606	50,595	133,934	133,896
AD ($S_{4}$)	25,827	25,818	50,606	50,595	133,934	133,896
DD ($S_{5}$)	25,827	25,818	50,606	50,595	133,934	133,896
AAA ($S_{6}$)	25,827	25,818	50,606	50,595	133,934	133,896
AAD ($S_{7}$)	25,827	25,818	50,606	50,595	133,934	133,896
ADD ($S_{8}$)	25,827	25,818	50,606	50,595	133,934	133,896
DDD ($S_{9}$)	25,827	25,818	50,606	50,595	133,934	133,896
AH ($S_{10}$)	25,827	25,817	50,606	50,594	133,934	133,897

A is the GRM of SNP additive values. D is the GRM of SNP dominance values. AA is the GRM of A × A values. AD is the GRM of A × D values. DD is the GRM of D × D values. AAA is the GRM of A × A × A values. AAD is the GRM of A× D × D values. ADD is the GRM of A × D × D values. DDD is the GRM of D × D× D values. AH is the GRM of haplotype additive values. $S_{i}$ (i = 1,…,10) is the multifactorial notation for the n×n genomic relationship matrix of the $i^{\mathrm{th}}$ effect type described in Materials and Methods.

**Table 6:** Five samples for genomic prediction and estimation for DPR.

	Sample 3 (Subset of Sample 1)	Sample 1	Sample 2	Sample 4 (Subset of Sample 2)	Sample 5 (Subset of Sample 2)
Number of cows	25,827	50,606	133,934	70,000	90,000
Number of SNPs	75,209	75,209	74,855	74,855	74,855
Purpose	Identifying the best haplotype model	Identifying the best epistasis model	Large sample analysis of prediction accuracy and heritability estimates	Additional evidence for the impact of sample size on prediction accuracy	Additional evidence for the impact of sample size on prediction accuracy

## Data Availability

The original genotype data are owned by third parties and maintained by the Council on Dairy Cattle Breeding (CDCB). A request to CDCB is necessary for obtaining data access on research, which may be sent to: João Dürr, CDCB Chief Executive Officer (joao.durr@cdcb.us). All other relevant data are available in the manuscript and Supplementary Materials.

## Supplementary Materials

The supporting information can be downloaded at: https://www.mdpi.com/article/10.3390/ijms26125687/s1.
